# Integrating MaxEnt and copula models to assess plague-associated ecological suitability under climate change in Northern Tianshan, China

**DOI:** 10.1371/journal.pntd.0014818

**Published:** 2026-10-26

**Authors:** Fang Li, Guoyu Zhao, Abulimiti Moming, Xiaobo Liu, Ping Ma, Shang Zhan, Huiqian Wang, Jianhui Chen, Wuliken Tuerdali, Qiguo Wang, Tao Luo, Haiyan Wu, Yujiang Zhang

**Affiliations:** 1 Xinjiang Key Laboratory of Vector-borne Infectious Diseases, Urumqi, Xinjiang Uygur Autonomous Region, China; 2 The Center for Disease Control and Prevention of Xinjiang Uygur Autonomous Region, Urumqi, Xinjiang Uygur Autonomous Region, China; 3 National Key Laboratory of Intelligent Tracking and Forecasting for Infectious Diseases, National Institute for Communicable Disease Control and Prevention, Chinese Center for Disease Control and Prevention, Chinese Academy of Preventive Medicine, Beijing, China; 4 The Center for Disease Control and Prevention of Yining County, Yining, Xinjiang Uygur Autonomous Region, China; Egerton University, KENYA

## Abstract

**Background:**

Plague, a paradigmatic zoonotic disease, depends on the intricate interplay among ecological environments, climatic conditions, and host-vector-pathogen interactions for its transmission. Climate change is reshaping the ecological distribution and transmission risks of plague. However, a systematic understanding of the future epidemiological trends for the Northern Tianshan Natural Plague Focus in Xinjiang remains limited.

**Methods:**

By employing an optimized maximum entropy model (MaxEnt) integrated with joint distribution Copula functions, the spatiotemporal dynamics of *Marmota baibacina*, *Yersinia pestis*, and plague transmission risk under both current and future climate change scenarios were evaluated.

**Results:**

The results revealed that the high-suitability habitats for both *M. baibacina* and *Y. pestis* within the focus were projected to exhibit trends of contraction and expansion in the future. These patterns could be attributed to the distinct environmental factors shaping their distributions. Specifically, the distribution of *M. baibacina* was primarily determined by temperature (Bio6 and Bio7) and precipitation (Bio15), whereas the distribution of *Y. pestis* was predominantly influenced by Bio15 and elevation. The spatial distribution of high-risk areas for plague transmission was projected to undergo dynamic changes, characterized by phases of stability, contraction, and expansion. Furthermore, the centroid of these high-risk regions may shift from Wusu City to Nileke County.

**Conclusions:**

Under future climate scenarios, the distribution and transmission risk of plague in the focus may be influenced by environmental factors, resulting in scenario- and period-dependent spatial changes. The western region of the focus is anticipated to experience the most pronounced shifts and is projected to remain at elevated risk of epizootic plague.

## Author summary

Plague, a deadly zoonotic disease and potential bioterrorism threat, remains a major risk to rural, resource-poor communities—especially as climate change reshapes its spread. We focused on the Northern Tianshan Mountain plague focus in China, one of the world’s most active, to understand how future climate shifts will affect disease transmission. Using an improved ecological modeling approach combined with statistical tools to characterize statistical dependence between the modelled suitability outputs, we mapped suitable habitats for *Yersinia pestis* and its main host (*Marmota baibacina*) under different climate scenarios. We found that climate change may drive temporal shifts in predicted high-risk areas, with the center of transmission moving between key regions. The western part of the study area may experience the most pronounced changes and maintain persistently high risk long-term. Importantly, we identified unrecognized high-risk zones (like Xinyuan County) that need surveillance. This approach may inform surveillance prioritization, subject to local validation..

## Background

Plague, caused by *Yersinia pestis* (Lehmann & Neumann), is a deadly infectious disease characterized by severe mortality and rapid transmission. Historically, it has triggered three devastating pandemics, resulting in approximately 200 million deaths worldwide [1]. Even today, plague remains a global health concern: under the WHO International Health Regulations (IHR, 2005), it is a notifiable disease that may constitute a PHEIC, while its causative agent, *Y. pestis*, is classified by the US CDC as a Category A bioterrorism agent [2]. This ongoing threat is exemplified by the 2017 urban pneumonic plague outbreak in Madagascar, with 2,417 confirmed, probable, and suspected plague cases reported by WHO through 26 November 2017 [3]. This incident highlights the persistent challenges in controlling such diseases, particularly in resource-limited settings, and underscores the ongoing limitations in understanding plague transmission mechanisms. As a natural focal disease, plague persistence is mediated by an ecological niche comprising biotic (e.g., rodent-flea-*Y. pestis* interactions) and abiotic factors (e.g., temperature, precipitation, elevation, vegetation, and soil) [4]. Genomic and ecological studies in Xinjiang further reveal that host-pathogen niche overlap and local environmental adaptation shape *Y. pestis* population structure and epizootic potential [5].

To date, a total of 15 distinct natural plague foci have been identified in China, five of which are in Xinjiang [6]. Long - term surveillance of *Y. pestis* has revealed that the natural plague focus of *Marmota baibacina* (Kastschenko) - *Spermophilus undulatus* (Pallas) in the northern slope of the Tianshan Mountains in Xinjiang, China, exhibits the most intense epizootic activity among all identified foci. Since the first officially recorded human plague outbreak in Xinjiang, 12 human plague incidences have been documented, with 11 (91.7%) occurring within this focus. Additionally, surveillance efforts from 1955 to 2020 yielded 1,847 *Y. pestis* strains from this region, accounting for 89.7% of all isolates (n = 2,058) [7].

The relationship between plague and ecological-geographical environments has long been a critical research topic in the field of natural focal diseases. Traditional studies have predominantly utilized statistical approaches, such as correlation analysis [8], principal component analysis [9], and cluster analysis [10], to explore these relationships. By examining biological factors (e.g., infected animals and vector fleas) and climate factors (e.g., temperature and precipitation), these studies have demonstrated that both biotic and abiotic factors are closely associated with the occurrence of plague. Moreover, the impacts of global climate change on ecological and geographical environments are intensifying. Recent ensemble species distribution models have projected climate-driven shifts in plague host ranges and epizootic risks across Central Asia [11,12], underscoring the urgency of region-specific risk forecasts. According to projections from the Intergovernmental Panel on Climate Change (IPCC) Sixth Assessment Report (AR6) [13], the global surface temperature is expected to rise by an average of 1.5–2°C by 2081–2100 compared to the pre-industrial era (late 19th century). This sustained increase in temperature may further alter the eco-geographical distribution of plague and exacerbate its transmission risks, highlighting the need for continued attention and in-depth research. In this study, an optimized MaxEnt model was employed, incorporating occurrence data of the primary host species, *M. baibacina*, and *Y. pestis* isolation points, to predict their suitable distribution areas under future climate change scenarios. Subsequently, conditional copula functions were utilized in this study to integrate the distribution probabilities output by the MaxEnt model, thereby quantifying the high - risk plague areas and elucidating their spatial distribution patterns within the focus.

## Materials and methods

### Consent for publication

We confirm that this manuscript represents the authors’ original work and has not been published previously in any form or language (partially or in full). The manuscript is not currently under consideration by any other journal, and all authors approve its submission to this journal.

### Study area

The plague natural focus of *M.baibacina* and *S.undulatus* in the northern Tianshan Mountains, stretching approximately 700 km from east to west, with altitudes ranging from 1,500–3,300 meters. This focus is characterized by a typical temperate continental arid climate [14], which results in significant annual temperature differences, low annual precipitation, and uneven distribution of rainfall. Specifically, the eastern region of the Northern Tianshan Mountains is relatively more humid, whereas the western region is drier. Currently, this focus has been successively detected in 10 counties and cities, including Urumqi County, Changji City, Hutubi County, Manas County, Shawan City, Wusu City, Jinghe County, Yining County, Wenquan County, and Nileke County.

### *M. baibacina* and *Y. pestis* occurrence records

Through a comprehensive review of historical literature, the precise geographic locations where *M. baibacina* was captured and *Y. pestis* was isolated in previous investigations were obtained. These sites were distributed across 10 endemic counties/cities. Subsequently, field surveys were conducted in these regions from July to September 2024. The locations where *M. baibacina* burrows or individuals were observed were marked as presence points for *M. baibacina*. Similarly, the locations where *Y. pestis* was isolated were designated as occurrence points for *Y. pestis*. All locations were georeferenced using globe positioning system (GPS) devices to record latitude and longitude coordinates.

The distribution points of *M. baibacina* and *Y. pestis* were systematically filtered and validated using the “ENMTools” package in R (version 4.4.2) to mitigate the risk of model overfitting and inflated performance estimates associated with spatial autocorrelation in species occurrence data. Ultimately, the initial dataset of 626 *M. baibacina* and 85 *Y. pestis* occurrence points was refined to 334 and 61 spatially independent points, respectively. These final datasets were used for subsequent species distribution modeling.

### Environmental factors

Nineteen bioclimate variables (S1 Table) from different periods at a resolution of 30 arc-seconds (1 km × 1 km) were obtained from the World Climate Database (https://www.worldclim.org; version 2.1). The periods were as follows: current (averages for 1970–2020), 2050s (averages for 2041–2060), 2070s (averages for 2061–2080), and 2090s (averages for 2081–2100). The future bioclimate variables were based on the shared socioeconomic pathway (SSP) scenario in the Coupled Model Intercomparison Project Phase 6 (CMIP6) and were simulated using the Beijing Climate Center Climate System Model 2 Medium Resolution (BCC-CSM2-MR). The study adopted SSP1-2.6, SSP2-4.5, SSP3-7.0, and SSP5-8.5 to represent low, medium, high, and higher-emission scenarios, respectively (S2 Table). Additionally, elevation variable (S1 Table) was obtained from the Resource and Environmental Science and Data Center of the Chinese Academy of Sciences (https://www.resdc.cn/) at a resolution of 30 arc-seconds (1 km × 1 km).

To mitigate model overfitting caused by autocorrelation and multicollinearity among environmental variables, jackknife analysis and spearman correlation analysis were employed. Initially, the jackknife analysis was employed to assess the initial contribution percentage of each environmental variable. Variables with a contribution rate exceeding 1% were retained for further analysis (S3 Table). Subsequently, the correlations among all variables were calculated. When the absolute value of Spearman’s rank correlation coefficient (|r|) between a pair of variables exceeded 0.90, the variable with the higher contribution percentage to the MaxEnt model was retained for subsequent analyses. The correlation coefficient matrix is presented (S1 Fig). Ultimately, six environmental variables, Isothermality (Bio3), Min temperature of coldest month (Bio6), Temperature annual range (Bio7), Precipitation seasonality (Bio15), Precipitation of warmest quarter (Bio18), and Elevation were selected for predicting the distribution of *M. baibacina*. For *Y. pestis*, five environmental variables were selected: Mean diurnal range (Bio2), Bio6, Bio7, Bio15, and Elevation. In addition, post-hoc variance inflation factor (VIF) assessments were conducted for the final retained variables, with results provided in S4 Table.

### Optimization of the MaxEnt model

MaxEnt (http://www.cs.princeton.edu/~schapire/maxent; v.3.4.1) was employed in this study to predict the potential suitable distributions of *M. baibacina* and *Y. pestis* across multiple time periods. The performance of the MaxEnt model was critically dependent on the regularization multiplier (RM) and feature combination (FC) [15], which collectively influenced the degree of model overfitting and its predictive accuracy. Generally, RM regulated model complexity, while FC determined the shapes of response curves. Parameter optimization was performed using the ENMeval package in R [16]. Specifically, eight levels of RM were set, ranging from 0.5 to 4.0 with an increment of 0.5. Additionally, six different FCs (L, LQ, H, LQH, LQHP, LQHPT; where L = linear, Q = quadratic, H = hinge, P = product, T = threshold) were used to optimize the MaxEnt model. Using the ENMevaluate function in the ENMeval package, all 48 possible parameter combinations were computed. The optimal model parameters were then selected based on the minimum Akaike information criterion(AIC), delta.AICc (delta.AICc = 0.00) [17].

### Model calibration and evaluation

The potential suitable distribution areas of *M. baibacina* and *Y.pestis* within the focus were predicted using the optimized MaxEnt model, incorporating the occurrence points of these species and the filtered environmental variables. 75% of the occurrence points were randomly selected to form the training set, while the remaining 25% constituted the testing set. The final predictions were derived from the average of 10 replicates for each model. The area under the receiver operating characteristic curve (AUC) serves as an effective indicator of model performance, with values ranging from 0 to 1 [18]. These values are commonly classified into five categories [19]: 0.0–0.6 (failure), 0.6–0.7 (poor), 0.7–0.8 (fair), 0.8–0.9 (good), and 0.9-1.0 (excellent).

### Classification of *M. baibacina* and *Y. pestis* habitats suitability

Following the classification criteria established by Cao et al. [20], the distribution possibility of *M. baibacina* and *Y. pestis* generated by MaxEnt modeling were categorized into four classes: very low suitability (*P* < 0.1), low suitability (0.1 ≤ *P* < 0.3), moderate suitability (0.3 ≤ *P* < 0.5), and high suitability (*P* ≥ 0.5).

### Calculation of plague transmission risk

Given that the distributions of *M. baibacina* and *Y. pestis* were not independent, the spread of the *Y. pestis* in nature typically relied on both its host and vector. The Copula function was capable of flexibly characterizing the dependence between two random variables, without being restricted by the distribution forms of these variables. The high-risk plague transmission area was defined by the conditional probability (*Y. pestis* habitats suitability ≥ 0.5 | *M. baibacina* habitats suitability ≥ 0.5), estimated through a bivariate conditional Copula function. The steps are as follows: (1) utilized the “raster”, “Vinecopula”, and “fitdistrplus” packages in R to import the distribution maps of *M. baibacina* and *Y. pestis*, formatted as  .asc files. (2) Missing values were handled using the “is.na ()” function from the “raster” package. Six common candidate marginal distribution models, including the normal, gamma, Weibull, log-normal, beta, and exponential distributions, were fitted. The best-fitting distribution was selected based on the minimum AIC value, which indicates the optimal balance between model fit and complexity. (3) The data were transformed into a uniform distribution based on the optimal marginal distributions. (4) The optimal Copula model was selected using the “BiCopSelect” function. (5) The conditional probability *(Y. pestis* habitats suitability ≥ 0.5 | *M. baibacina* habitats suitability ≥ 0.5) was calculated. (6) The high-risk areas for plague transmission were remapped to a raster format. The “ggplot2” package in R was used for visualization.

### Analysis of spatial pattern dynamics and centroid shift in the adaptation area

Initially, the plague transmission risk areas within the epidemic focus were categorized into high-risk and non-high-risk regions using the natural breaks classification method in ArcGIS (version 10.3), with a threshold of *P* = 0.5. Subsequently, spatial overlay analysis was employed to precisely delineate the spatial change types of the high-risk areas, which were classified into stable areas, contraction areas, and expansion areas.

Furthermore, a centroid shift analysis of plague transmission high-risk regions was conducted under different future climate change scenarios relative to the current period. Specifically, the “Centroid Changes (Lines)” tool in the SDMToolbox package of ArcGIS was employed. The geometric center of the contemporary plague transmission high-risk area was used as the reference point to predict the trajectory of geometric center changes in different future periods. Additionally, the standard deviation ellipse of the high-risk areas was calculated for each climate change scenario.

### Statistical analysis

Occurrence records of *M. baibacina* and *Y. pestis*, areas of high-risk regions, and range of environmental variables were processed in Microsoft Excel 2016. The relationships between environmental factors and the highly suitable distribution areas of *M. baibacina* and *Y. pestis* were analyzed using generalized additive models (GAMs). Spatial contraction and expansion differences of high-risk plague transmission regions between eastern and western plague-endemic regions were compared using Student’s t -tests (normally distributed variables) or Kolmogorov-Smirnov tests (non-normally distributed variables), with all analyses performed in R.

## Results

### Evaluation of model performance

For distribution modeling using MaxEnt, the default parameters (RM = 1.0, FC = LQHP) yielded delta.AICc values of 92.28 for *M. baibacina* and 92.39 for *Y. pestis*. After optimization, *M. baibacina*’s model achieved optimal performance with RM = 1.0 and FC = LQHPT (delta.AICc = 0.00; Fig 1a), while *Y. pestis* required RM = 3.0 and FC = H (delta.AICc = 0.00; Fig 1b). Both models demonstrated predictive accuracy, with training AUC values of 0.953 ± 0.002 (for *M. baibacina*) and 0.962 ± 0.009 (for *Y. pestis*) under current conditions. Model robustness was maintained across all future climate scenarios, with mean AUC values consistently exceeding 0.950 (S5 Table), confirming their reliability for subsequent analyses.

**Fig 1:**
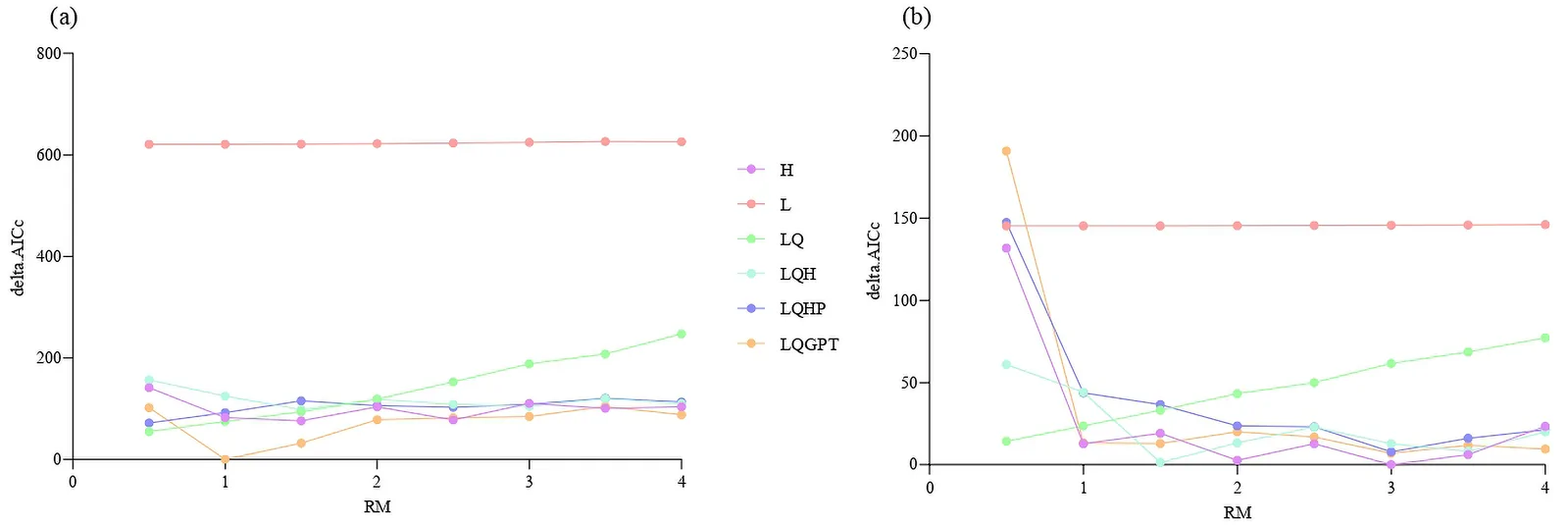
Output of MaxEnt models optimization using different combinations of regularization multipliers and feature classes. **(a)** *Marmota baibacina*; **(b)** *Yersinia pestis*.

### Current habitats distribution of *M. baibacina* and *Y. pestis*

The optimized MaxEnt model projected the potential distributions of both *M. baibacina* and *Y. pestis* habitats under current conditions, categorized into very low, low, moderate, and high suitability classes (Figs 2 and 3). For *M. baibacina*, moderate-to-high suitability areas formed a continuous belt along the Tianshan Mountain Range, covering all plague-endemic counties/cities and extending unexpectedly to the non-endemic Xinyuan County, the total area was 1.71 × 10^4^ km^2^. Similarly, *Y. pestis* distribution largely coincided with its primary host, *M. baibacina*, with moderate-to-high suitability areas encompassing both endemic regions and Xinyuan County, the total area was 2.25 × 10^4^ km^2^.

**Fig 2:**
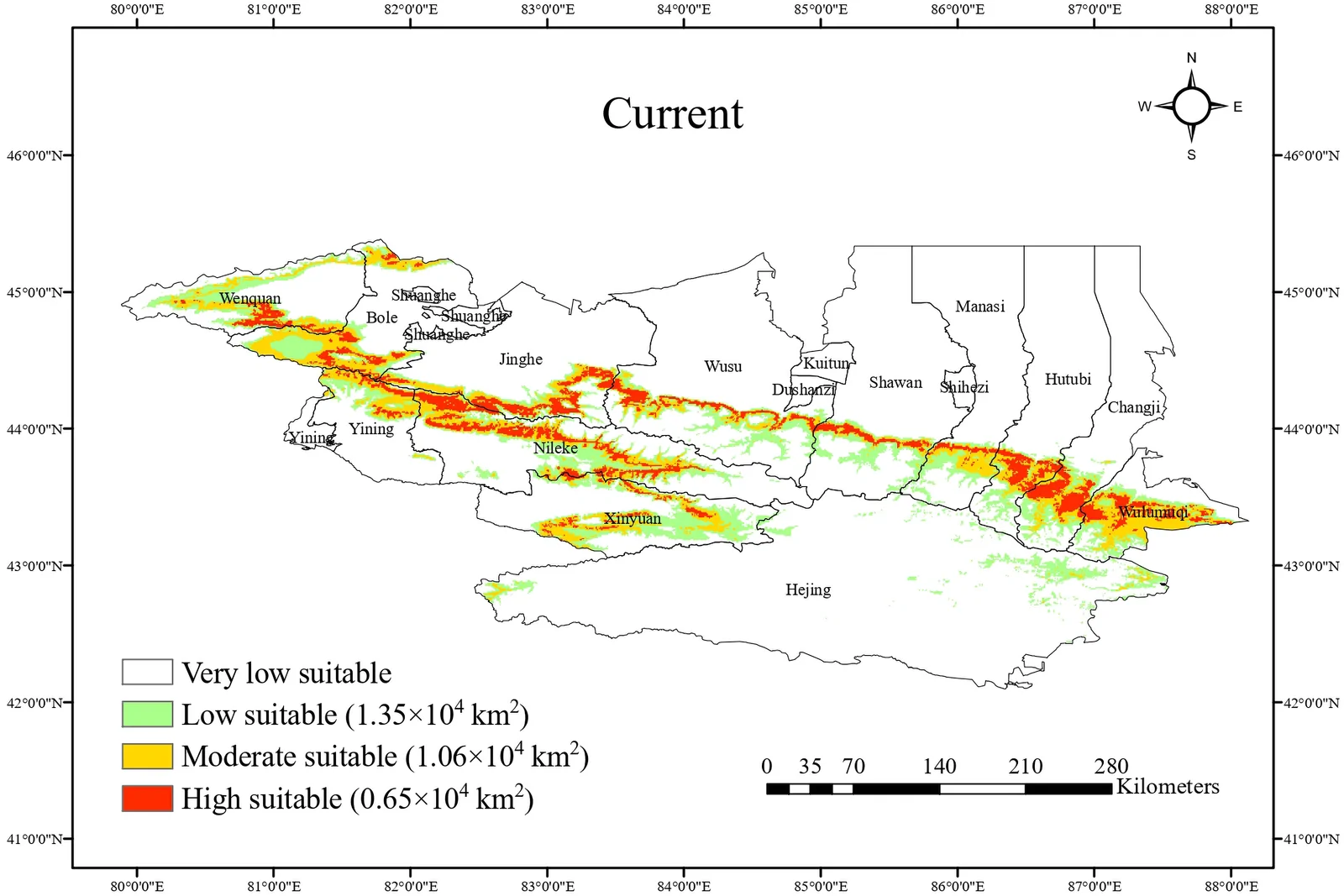
The potential distribution of *Marmota baibacina* under current conditions. The map was created in ArcGIS 10.3 software (ESRI Inc., Redlands, CA, USA). The source of the base layer shapefiles from the open access platform National platform for Common Geospatial Information Services (www.tianditu.gov.cn).

**Fig 3:**
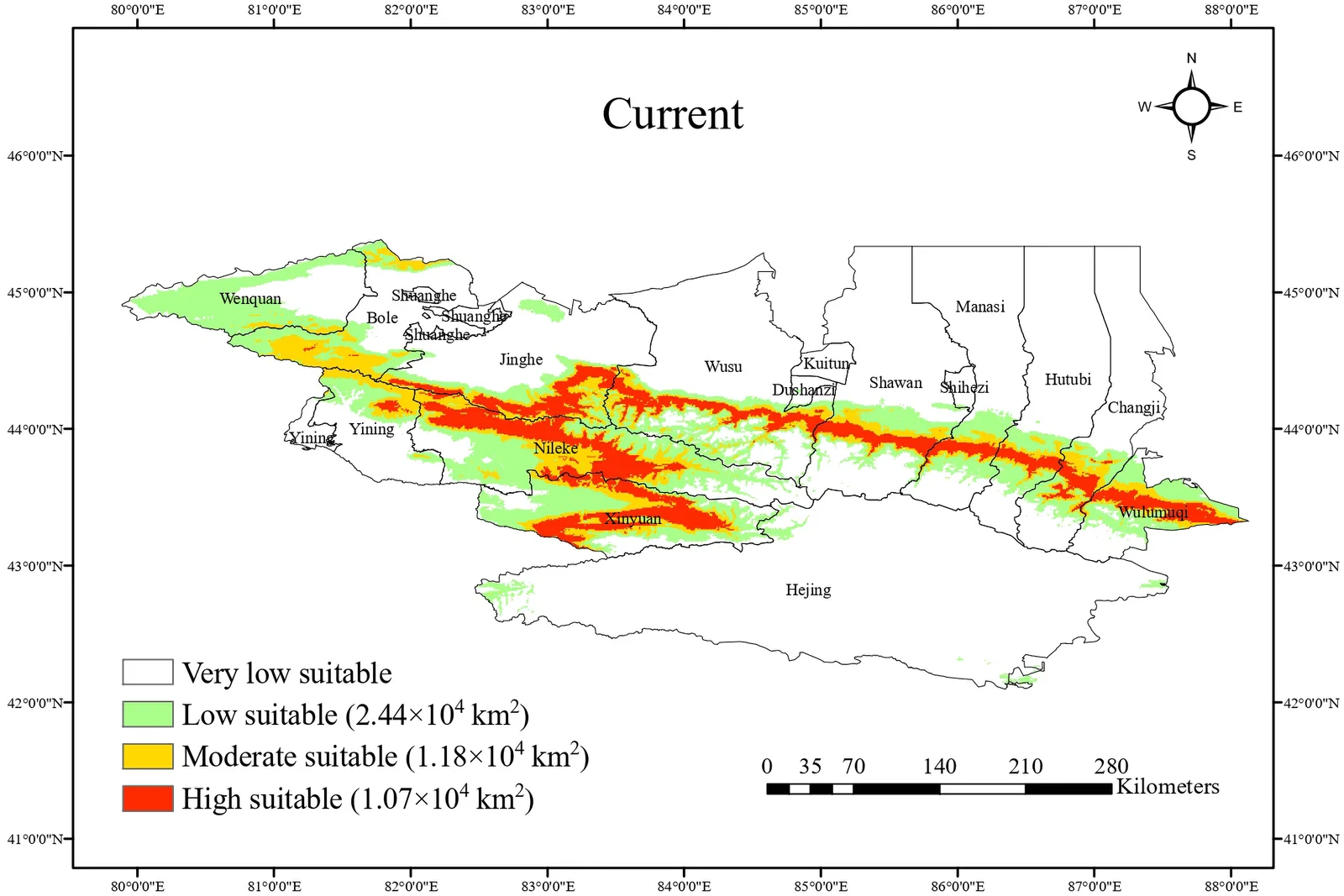
The potential distribution of *Yersinia pestis* under current conditions. The map was created in ArcGIS 10.3 software (ESRI Inc., Redlands, CA, USA). The source of the base layer shapefiles from the open access platform National platform for Common Geospatial Information Services (www.tianditu.gov.cn).

### Distribution prediction for *M. baibacina* and *Y. pestis* under future climate change scenarios

The optimized MaxEnt model projected the potential distribution of *M. baibacina* habitats across three time periods (2050s, 2070s, and 2090s) under four emission scenarios: low (SSP1-2.6), medium (SSP2-4.5), high (SSP3-7.0), and higher (SSP5-8.5), as presented in S2 Fig. The results indicated consistent habitat (including low, moderate, and high suitability areas) contraction for *M. baibacina* across all future periods and emission scenarios, except in the 2050s and 2090s under the medium-emission scenario (SSP2-4.5), where a slight expansion was observed relative to the current period. The contraction pattern was characterized by range shifts toward mountain interiors, with more pronounced losses in western regions (including Wenquan, Yining, Nileke, Jinghe, Wusu, and Xinyuan counties/cities) than in eastern regions (including Shawan, Manas, Hutubi, Changji, Hejing, and Urumqi counties/cities). The spatiotemporal shifts in high-suitability habitats were examined (Fig 4). Under the low-emission scenario (SSP1-2.6), the high-suitability habitat area was projected to decline consistently from the current 0.65 × 10^4^km^2^ to 0.43 × 10^4^km^2^ (2050s), 0.43 × 10^4^km^2^ (2070s), and 0.37 × 10^4^km^2^ (2090s), representing decreases of 33.14%, 33.14%, and 42.50%, respectively. Conversely, under the medium-emission scenario (SSP2-4.5), this area was projected to expand to 1.00 × 10^4^km^2^ (2050s), 0.71 × 10^4^km^2^ (2070s), and 1.32 × 10^4^km^2^ (2090s), corresponding to substantial increases of 53.46%, 9.92%, and 103.79%, respectively. The other two emission scenarios exhibited alternating contraction-expansion trend. Under the high-emission scenario (SSP3-7.0), the area was projected to initially expand to 0.69 × 10^4^km^2^ (a 5.42% increase) by the 2050s, then contract to 0.42 × 10^4^km^2^ (a 35.16% decrease) in the 2070s, and further decline to 0.50 × 10^4^km^2^ (a 22.96% decrease) by the 2090s. Under the higher-emission scenario (SSP5-8.5), the area was projected to first contract to 0.60 × 10^4^km^2^ (a 7.35% decrease) in the 2050s, then expand to 0.80 × 10^4^km^2^ (a 22.90% increase) in the 2070s, before finally declining to 0.38 × 10^4^km^2^ (a 41.57% decrease) by the 2090s.

**Fig 4:**
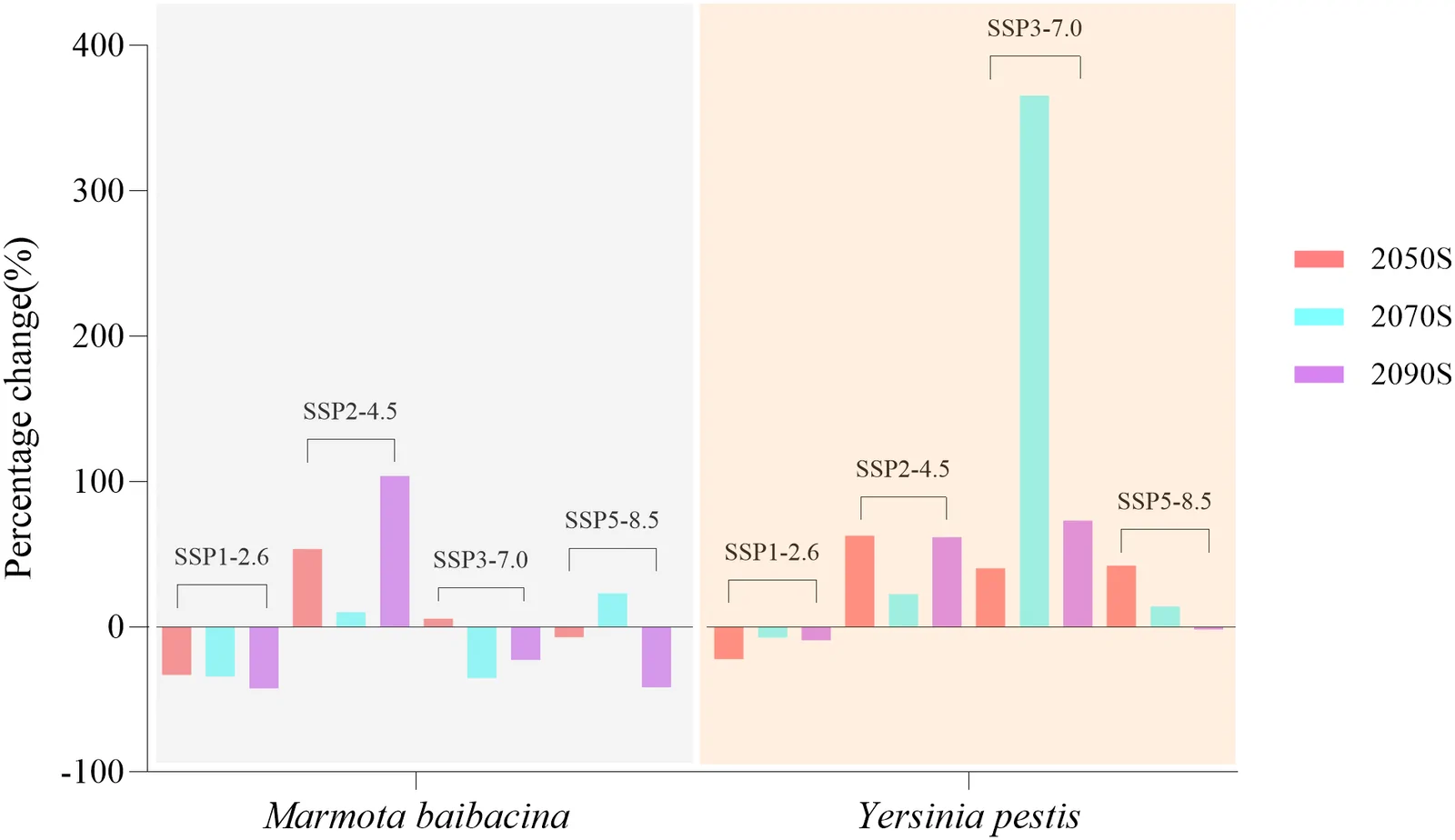
Percentage changes in the high-suitability habitats of *Marmota baibacina* and *Yersinia pestis* under climate change scenarios, relative to current conditions.

Similarly, the potential distribution of *Y. pestis* in the focus under future climate change scenarios were analyzed (S3 Fig). Results demonstrated an overall expansion of suitable habitats for *Y. pestis* (including low, moderate, and high suitability areas) compared to current baseline. Additionally, Fig 4 illustrated the dynamics of *Y. pestis* high-suitability habitats. Under the low-emission scenario (SSP1-2.6), the high-suitability habitats for *Y. pestis* were projected to contract to 0.83 × 10^4^km^2^ (2050s), 0.99 × 10^4^km^2^ (2070s), and 0.97 × 10^4^km^2^ (2090s), representing decreases of 22.43%, 7.48%, and 9.35% relative to current area (1.07 × 10^4^km^2^), respectively. In contrast, the other scenarios exhibited expansions, with areas averaging 1.59 × 10^4^km^2^, 2.77 × 10^4^km^2^, and 1.27 × 10^4^km^2^ under the medium - (SSP2-4.5), high - (SSP3-7.0), and higher - (SSP5-8.5) emission scenarios, corresponding to increases of 48.60%, 158.88%, and 18.69%, respectively. The peak expansion occurs under the high-emission scenario (SSP3-7.0).

### The key environmental variables influencing *M. baibacina* and *Y. pestis*

The contribution rates of six environmental variables revealed distinct drivers influencing the distribution of *M. baibacina* under future climate change scenarios (Fig 5a). Under the current conditions, three dominant factors were the Min temperature of coldest month (Bio6), Temperature annual range (Bio7), and Precipitation seasonality (Bio15), collectively explaining >60% of the total relative contribution, and maintained their dominant influence across all climate change scenarios. S6 Table presented the changes in the three key environmental factors influencing the highly suitable habitat of *M. baibacina* under future climate change scenarios compared to current conditions. Bio6 values fluctuated near the current level (-22.12°C), ranging from -22.16°C to -21.80°C (mean ± SD: -22.00 ± 0.12°C); Bio7 values showed consistent declines (range: 41.06°C to 41.76°C; mean ± SD: 41.56 ± 0.1°C); and Bio15 values exhibited fluctuations (range: 73.78 to 74.36; mean ± SD: 74.20 ± 0.15). The Generalized Additive Model (GAM) results (Fig 6a) revealed a significant nonlinear effect of Bio15 on the highly suitable habitat area for *M. baibacina* (edf = 1.895, *F* = 6.221, *P* = 0.021), with the model explaining 74.84% deviance. When Bio15 was below 74.14, the habitat area decreased with increasing Bio15 values, whereas above this threshold (≥ 74.14), the area exhibited a positive response to further Bio15 increases. In contrast, the other two examined factors (Bio6: F = 0.686, *P* = 0.413; Bio7: F = 2.538, *P* = 0.150) showed no statistically significant relationships with habitat suitability.

**Fig 5:**
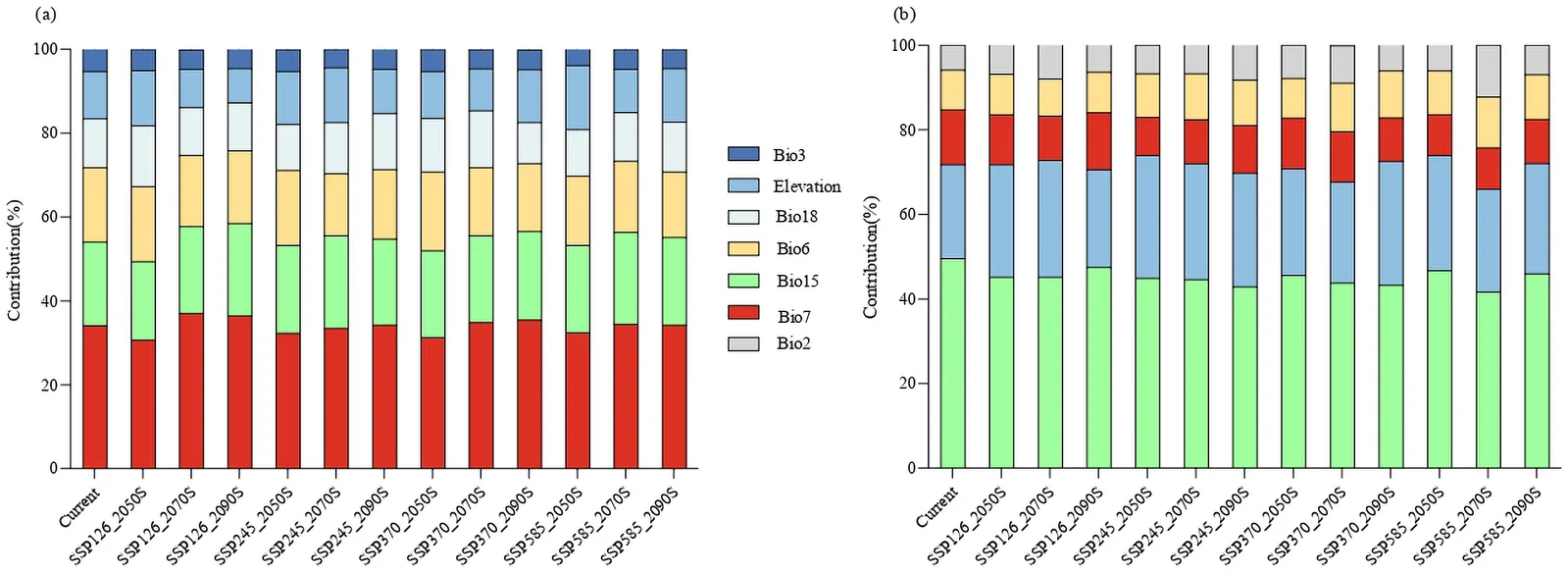
Key environmental variables identified distinct drivers influencing the distribution of *Marmota baibacina* and *Yersinia pestis* under climate change scenarios. **(a)** *Marmota baibacina*; **(b)** *Yersinia pestis*.

**Fig 6:**
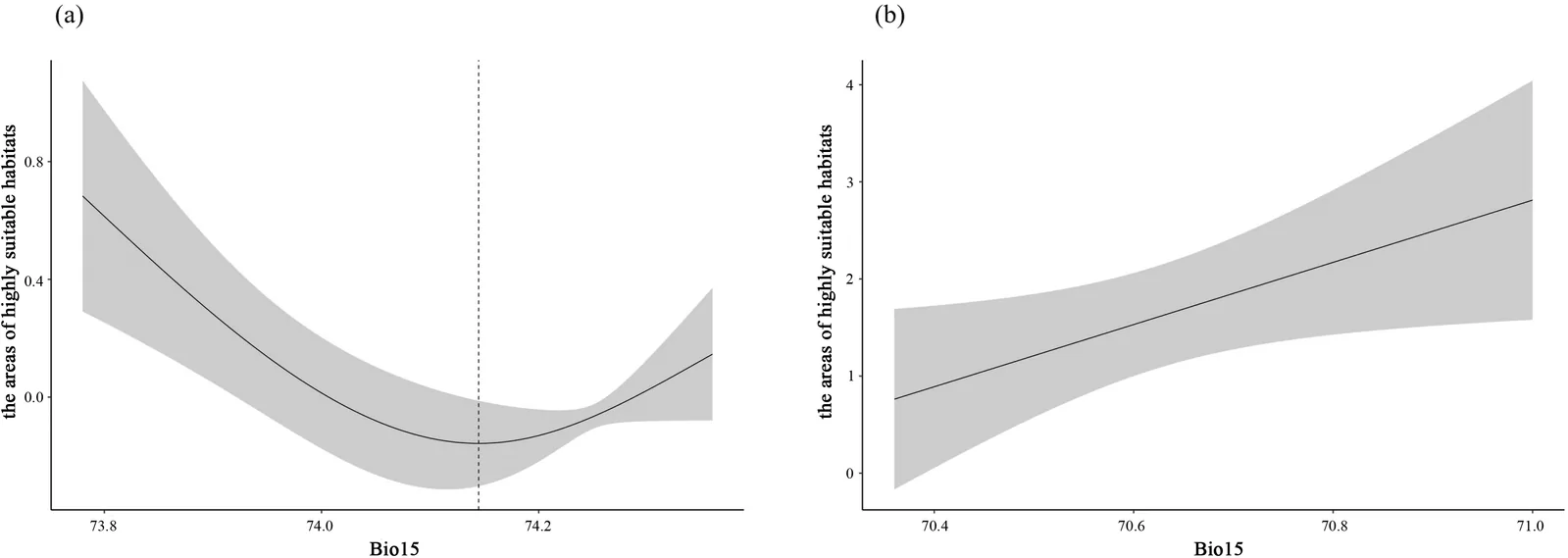
Relationship between the predicted high-suitability habitat distribution of (a) *Marmota baibacina* and (b) *Yersinia pestis* and Precipitation Seasonality (Bio15) in the focus.

Similarly, for *Y. pestis*, five environmental variables were identified as key drivers influencing its distribution under future climate change scenarios (Fig 5b). Of these, Bio15 and elevation were the two dominant factors, collectively accounting for > 60% of the total relative contribution under current conditions and maintaining their dominant influence across all future scenarios. Under future climate change scenarios (S6 Table), Bio15 was projected to fluctuate only slightly, remaining very close to its current value of 70.62, with a range between 70.36 and 71.00 (mean ± SD: 70.62 ± 0.20). Elevation followed a similar trend, varying from 1,888.45m to 1,946.84 m (mean ± SD: 1,921.04 ± 16.31 m). GAM results (Fig 6b) revealed that Bio15 exhibited a weak but significant positive correlation with the area of highly suitable habitats for *Y. pestis* (*β* = 3.20, 95%*CI* [0.402-5.999], *P* = 0.047), indicating that each unit increase in Bio15 corresponded to an average habitat expansion of 3.2 units, with the model explaining 31.35% deviance. In contrast, elevation demonstrated no statistically significant relationship with habitat suitability (*P* = 0.729).

### High-risk areas for plague transmission under current and future climate change scenarios

The results of Copula function showed that under current conditions, the high-risk areas for plague transmission in the focus were primarily distributed in a continuous belt across Urumqi County, Changji City, Hutubi County, Manas County, Shawan City, Wusu City, Jinghe County, Nileke County, and Xinyuan County (S4 Fig). Under future climate change scenarios, the spatial pattern of high-risk plague transmission area was projected to shift, followed a trend of stability, contraction, and expansion (S4 Fig). Specifically, under the low and medium-emission scenarios (SSP1-2.6 and SSP2-4.5), the high-risk area for plague transmission remained consistently reduced across all three periods (Fig 7), with mean areas of 0.44 × 10^4^km^2^ and 0.45 × 10^4^km^2^, respectively. This represented a decrease of 8.33% and 6.25% compared to the current area (0.48 × 10^4^km^2^). Conversely, under the high and higher - scenarios (SSP3-7.0 and SSP5-8.5), the high-risk area increased to an average of 0.51 × 10^4^km^2^ and 0.55 × 10^4^km^2^, corresponding to an increase of 6.25% and 14.58% relative to the current baseline (Fig 7). Further analysis revealed that the contraction and expansion dynamics of this plague focus were primarily concentrated in the western region (S7 Table). The western region exhibited significantly greater distributional dynamics than the eastern region. This disparity was characterized by both higher contraction (Kolmogorov-Smirnov *Z* = 2.449, *P* < 0.001) and greater expansion (Student’s t-test, *t* = 5.024, *P* < 0.001), as presen*t*ed in S5 Fig. Further analysis of spatial dynamics in western high-risk areas identified three focal counties/cities (Nileke, Jinghe, and Wusu) as hotspots of spatial change (S6 Fig). Under all future climate change scenarios, these hotspots account for over 50% of the total spatial change (both expansion and contraction) in the western region.

**Fig 7:**
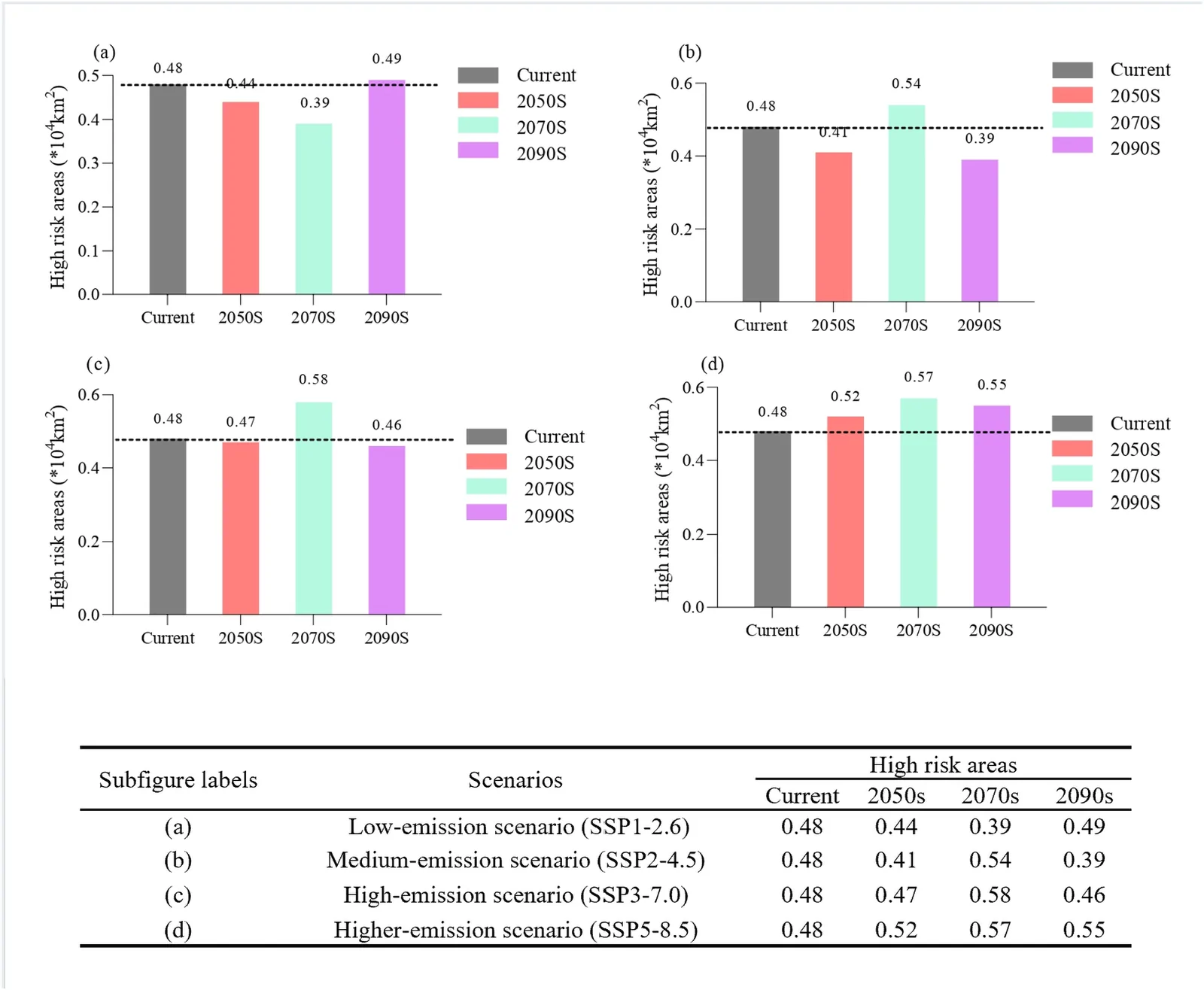
The focus areas of high plague transmission risk under current and future climate scenarios. **(a)** Low-emission scenario (SSP1-2.6); **(b)** Medium-emission scenario (SSP2-4.5); **(c)** High-emission scenario (SSP3-7.0); **(d)** Higher-emission scenario (SSP5-8.5).

### Geographical shifts in the centroid of high-risk plague transmission areas under future climate change scenarios

Centroid analysis revealed distinct migration patterns of high-risk plague transmission areas between Wusu City and Nileke County (Fig 8). Under the low-emission scenario (SSP1-2.6), the centroid shifted southwestward along the Wusu-Nileke axis. Under the medium and high-emission scenario (SSP2-4.5 and SSP3-7.0), the centroid showed northwest-eastward trajectories ultimately returning northwest, following a path from Wusu City to Nileke County and back to Wusu City. Under the higher-emission scenario (SSP5-8.5), the centroid first shifted northwestward, then eastward, and finally moved southwestward, following a path from Wusu City to Nileke County and back to Wusu City.

**Fig 8:**
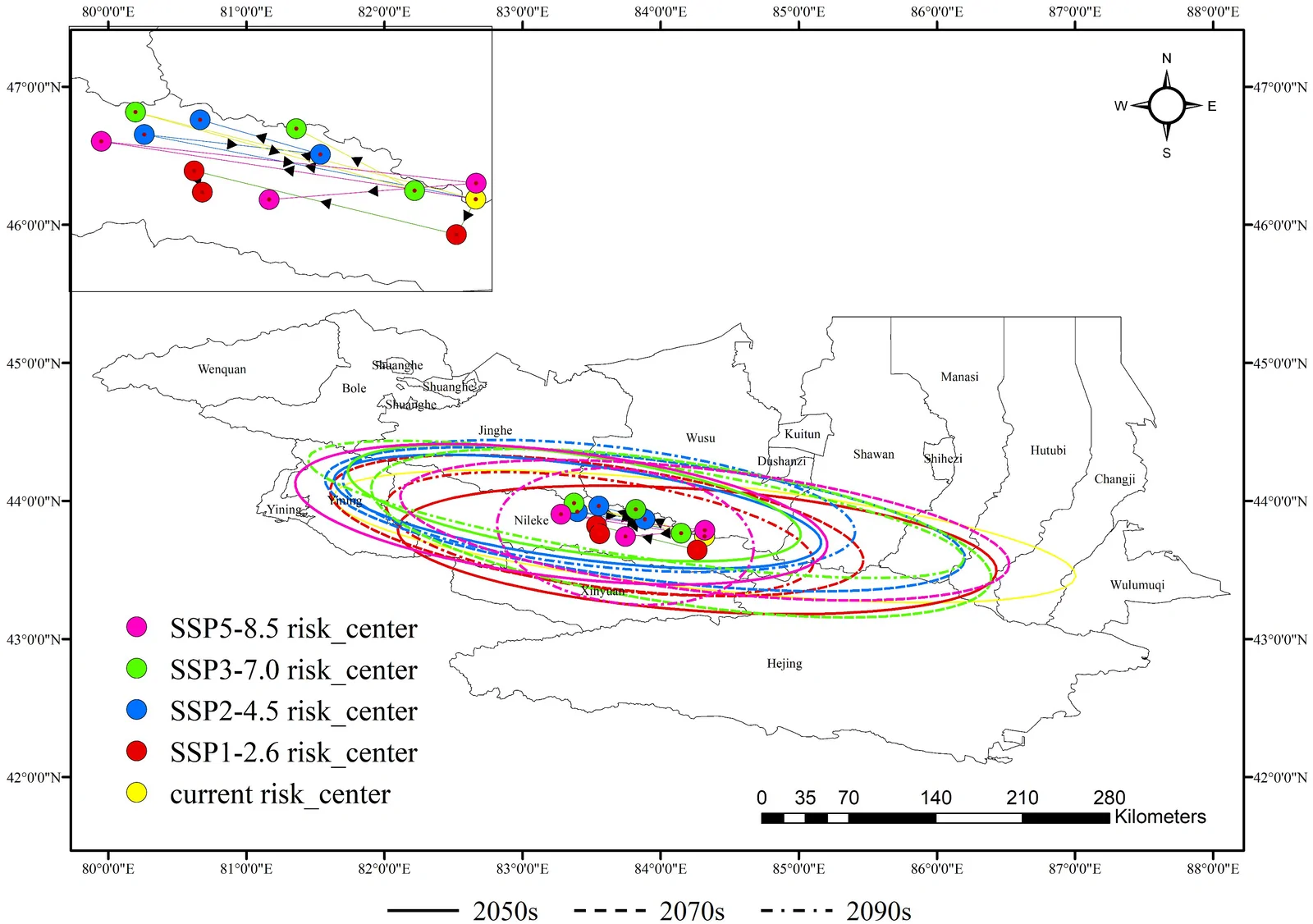
Geographical distribution changes in the centroid of the suitable growing area of high-risk plague transmission under climate change scenarios. The map was created in ArcGIS 10.3 software (ESRI Inc., Redlands, CA, USA). The source of the base layer shapefiles from the open access platform National platform for Common Geospatial Information Services (www.tianditu.gov.cn).

Meanwhile, the standard deviation ellipse analysis of the high-risk plague transmission areas indicated that 68% of these areas were primarily concentrated in the central region of the epidemic source area.

## Discussion

Plague, as a typical zoonotic disease, spread in nature through complex interactions among ecosystems, climate, and host-vector-pathogen dynamics. Empirical studies have demonstrated that climate change modulated the survival cycle of *Y. pestis*, the reproductive strategies of rodent hosts, and the maturation rate of vector fleas, triggering cascading effects among hosts, vectors, and pathogens. These ecological perturbations were fundamentally reshaping plague transmission risk distribution patterns. In this context, ecological niche models, particularly the Maximum Entropy (MaxEnt) model, have emerged as pivotal technical instruments for elucidating the climate-plague coupling mechanism and forecasting transmission risks proactively, owing to their capability for precise quantification of environmental thresholds and species distributions.

Up to date, the Maxent model has gained prominence in disease ecology for predicting species distributions, elucidating risk factors, and quantifying transmission risks in zoonotic disease systems [20,21]. However, the computationally intensive calibration process has led to prevalent over reliance on default parameters in many studies [21,22], potentially resulting in overfitting, suboptimal variable selection, and compromised model evaluation. In this study, the ENMeval package in R was employed [23,24] to systematically evaluate model performance across parameter combinations, identifying optimal settings through comprehensive calibration. Moreover, Copula function was employed for the first time the to quantify high-risk areas for plague transmission within the focus. As a powerful statistical tool, the Copula function was capable of precisely modeling and analyzing complex dependency relationships among multiple variables, particularly excelling in capturing nonlinear dependencies [25]. Currently, the Copula function was predominantly utilized in fields such as finance [26], hydrology [27], and other fields [28]. For instance, Fang et al. proposed a bivariate Copula conditional probability model to simulate the likelihood of vegetation degradation under multiple drought scenarios in the Loess Plateau region of China [27]. Similarly, the transmission dynamics of zoonotic diseases typically involved complex interactions among multiple factors. Given these intricate dependencies, the Copula function emerged as a particularly suitable analytical tool, as it could effectively integrate multivariate relationships. This approach consequently held significant potential for advancing zoonotic disease research, particularly in enhancing early warning systems, improving risk assessment accuracy, and informing targeted control measure development. Application of this method identified nine high-risk counties/cities within the focus, with Xinyuan County as the sole exception (a non-endemic area), and the remaining eight classified as active endemic regions. These findings showed high concordance with historically documented human plague outbreak areas in the focus, confirming the method’s reliability.

This study suggested that the high-risk areas for plague transmission were consistently larger in the western regions than in the eastern regions across all time periods and climate scenarios (Student’s t-test, *t* = 4.862, *P* < 0.001; see S7 Table). Moreover, the study also found that under future climate scenarios, the distribution and prevalence of plague in the western region exhibit more pronounced fluctuations compared to the eastern region, with high-risk areas persistently existing in the western region over the long term (S4 Fig). This phenomenon may be closely related to the high genetic diversity of *Yersinia pestis* genomes in the region, as discovered by Zhang et al [29], who reported found that higher *Y. pestis* genetic diversity in western strains (3 DFR genotypes vs. 1 in the east). Research by Cui et al [30] on the genomic evolution of *Yersinia pestis* in this region also confirmed that changes in local climatic conditions have led to genomic mutations in the bacterium corresponding to periods of climate change. These mutations have altered the efficiency of plague transmission by fleas, thereby potentially contributing to variations in plague prevalence. Combined with the findings of Ma et al [31], who demonstration that genomic diversity positively correlates with human plague outbreak frequency, we hypothesized that this distribution pattern under future climate scenarios in the region may be the combined result of the adaptation of *Yersinia pestis,* its hosts, and vectors to future climatic conditions.

Notably, this study identified previously unrecognized high-risk plague transmission regions in Xinyuan County, a region not traditionally classified as plague-endemic. Located within the Ili River Valley, Xinyuan County spanned elevations of 1,800–3,700 m and featured a mid-temperate humid continental climate, with annual precipitation of approximately 700–900 mm and mountain meadow steppe vegetation. Previous studies have reported the occurrence of *M. baibacina* in northern Xinyuan County [32], which bordered Nileke County—a known plague-endemic region. Given the absence of natural geographical barriers between these areas, the region has long been considered a potential region for animal plague, yet no *Y. pestis* surveys had been conducted. Similarly, other suspected plague areas, such as the Junggar Basin plague focus (2005) and the Altay Mountain plague focus (2021), were only confirmed as natural foci after prolonged investigations [33,34]. Our predictive modeling of *M. baibacina* distribution in this region showed complete concordance with historical survey data, while the predicted *Y. pestis* distribution revealed previously unrecognized high-risk plague areas. These findings underscored the necessity for implementing systematic plague surveillance programs in these regions and incorporating plague risk assessment into comprehensive monitoring systems.

Numerous studies have confirmed that climate and topography are the dominant drivers of plague transmission [35–37]. In this natural plague focus, precipitation has been identified as a key environmental factor influencing the prevalence of animal plague and the genetic variation of *Y. pestis*. Sun et al [38]. reported a biphasic effect of precipitation (initial promotion followed by suppression) on plague prevalence, exhibiting a certain time lag. Furthermore, Cui et al [30]. demonstrated that precipitation deficit significantly elevates the mutation frequency in the rpoZ gene among local *Y. pestis* strains. Building upon these findings, our study identified Bio15 as a pivotal factor governing the sustainability of suitable habitats for *M. baibacina* and *Y. pestis*. Notably, *M. baibacina* exhibited significantly broader tolerance to Bio15 than *Y. pestis* (Student’s t-test, *t* = 54.42, *p* < 0.001). Ecological adaptations explained this divergence: *M. baibacina* tolerated precipitation variability through burrowing (buffering microclimate) and dietary shifts (herbs to roots/insects). Conversely, *Y. pestis* was constrained by strict humidity requirements (both excessively high and low humidity accelerate its death) and vector-borne transmission bottlenecks, restricting it to narrower climatic conditions [39]. This climatic niche segregation partially explained why plague outbreaks remain localized despite the host’s widespread distribution in natural foci. Additionally, this study elucidated how Bio15 differentially influenced the highly suitable habitats of *M. baibacina* and *Y. pestis*. For *M. baibacina*, Bio15 exhibited a nonlinear threshold effect (74.14): when Bio15 < 74.14, increased variability constrained habitat expansion, but beyond this threshold, the effect became facilitative. In contrast, Bio15 had a gradual linear effect on *Y. pestis*, with suitable range expanding as Bio15 increased. These findings represented a significant advance beyond conventional studies limited to mean annual precipitation, offering novel eco-epidemiological insights into climate variability’s dual regulatory role in plague transmission dynamics. We proposed integrating precipitation seasonality as an essential parameter in plague risk assessment frameworks to optimize early-warning systems and targeted intervention strategies.

Finally, this study identified Nileke, Jinghe, and Wusu counties/cities as hotspots for high-risk areas of spatial change. This finding was highly consistent with historical surveillance data. Between 1955 and 2009, a total of 1,336 *Y. pestis* strains were isolated from these three areas, accounting for 79.95% (1,336/1,671) of all isolates obtained from this plague focus [40]. These data further confirmed their epidemiological significance as core plague focus. Additionally, this study further predicted that the center of high-risk plague transmission areas would migrate between Wusu City and Nileke County in the future. Cui Yujun et al. [30] discovered that *Y. pestis* strains isolated from the Wusu city harbored a missense mutation in the rpoZ gene, exhibiting significantly accelerated biofilm formation compared to wild-type strains (F = 161.48, *P* < 0.001). This mutation likely enhanced environmental persistence and transmission risks in Wusu by promoting extracellular polysaccharide production, thereby increasing bacterial colonization capacity and survival fitness.

This study had certain limitations. While the bivariate conditional Copula framework integrating *M.baibacina* and *Y.pestis* habitat suitability effectively quantifies plague epizootic risk, this modeling pipeline adopts a simplified ecological premise: the spatial distribution of flea vectors (the critical transmission medium for *Y. pestis*) is implicitly assumed to fully co-occur with areas where both host and pathogen attain high suitability (suitability ≥ 0.5). This simplification overlooks independent climate-driven shifts in flea survival, reproduction, and dispersal that may decouple vector ranges from host–pathogen co-suitable zones under future global warming. Climate change could alter microclimatic humidity, surface temperature, and vegetation litter conditions that exclusively regulate flea population viability, creating mismatches between modeled high-risk zones and actual plague transmission hotspots where competent flea vectors are absent. Future iterations of this risk assessment framework should incorporate flea occurrence and niche data to resolve this structural limitation and refine predictive precision.

## Conclusions

This study not only provided model-based evidence to inform surveillance prioritization for plague prevention and control strategies but also introduced a novel methodological framework for evaluating the transmission risks of zoonotic diseases in the era of global climate change. Combined with the spatial-temporal variation patterns of plague transmission risk and key environmental thresholds identified in this study, we propose targeted, operable prevention and surveillance strategies for local public health and plague control agencies. First, systematic year-round passive plague surveillance could be considered following local assessment in Xinyuan County, a model-identified potential surveillance priority outside traditional endemic areas intensive active field flea sampling and marmot burrow monitoring could be considered following local assessment annually from May to September, the peak activity period of hosts and vectors. Second, we explore a climate-based early warning indicator for plague risk: in the western core of the plague focus, a model-derived the precipitation seasonality index (Bio15) threshold of 74.14 may warrant further evaluation as a potential trigger; if locally validated, rodent population density surveys could be considered at monthly rather than quarterly frequency to capture early epizootic signals. Third, limited plague surveillance human and material resources should be prioritized to the three core administrative units in the western plague focus identified in this study (Nileke County, Jinghe County, and Wusu City). Under all climate emission scenarios,these three units account for over 50% of the total spatial change—that is, the combined expansion and contraction—of plague high-risk areas. Historical pathogen isolation data also support them as the core epizootic hotspots of the Northern Tianshan plague focus. Long-term fixed monitoring stations and emergency response teams may need to be deployed in these three regions to cope with the projected shifts across scenarios and periods of plague risk centroids between Wusu and Nileke under future climate change. Collectively, this multi-layered surveillance and early warning framework can help health departments allocate limited prevention resources efficiently, improve surveillance targeting, and proactively respond to climate-driven plague risk fluctuations in the Northern Tianshan Mountains, although these potential efficiency gains require evaluation.

## Supporting information

S1 Table Environmental variables used in the model for *Marmota baibacina* and *Yersinia pestis.* (PDF)

S2 Table Four emission scenarios in the shared socioeconomic pathway scenario. (PDF)

S3 Table Percentage contribution of 20 environmental variables in the MaxEnt model for *Marmota baibacina* and *Yersinia pestis.* (PDF)

S4 Table Post-hoc variance inflation factor (VIF) assessment of environmental variables following Spearman-based selection for *M. baibacina* and *Y. pestis* MaxEnt models. (PDF)

S5 Table Average AUC values of training data for MaxEnt models optimized under current and future climate change scenarios. (PDF)

S6 Table The changes of key environmental factor in regions with high suitable habitats for *Marmota baibacina* and *Yersinia pestis* under future climate change. (PDF)

S7 Table The areas of potential distribution regions with high transmission risk for plague under current and future climate conditions in the focus. (PDF)

S1 Fig Correlation coefficient matrix of 20 environmental variables. (a) *Marmota baibacina* and (b) *Yersinia pestis*.
(TIF)

S2 Fig Predicted potential habitat of *Marmota baibacina* in three time periods (2050s, 2070s, and 2090s) under four climate change scenarios (SSP1-2.6, SSP2-4.5, SSP3-7.0, and SSP5-8.5). The map was created in ArcGIS 10.3 software (ESRI Inc., Redlands, CA, USA). The source of the base layer shapefiles from the open access platform National platform for Common Geospatial Information Services (www.tianditu.gov.cn).
(TIF)

S3 Fig Predicted potential habitat of *Yersinia pestis* in three time periods (2050s, 2070s, and 2090s) under four climate change scenarios (SSP1-2.6, SSP2-4.5, SSP3-7.0, and SSP5-8.5). The map was created in ArcGIS 10.3 software (ESRI Inc., Redlands, CA, USA). The source of the base layer shapefiles from the open access platform National platform for Common Geospatial Information Services (www.tianditu.gov.cn).
(TIF)

S4 Fig The spatiotemporal shifts of high-risk plague transmission areas within the focus under current and climate change scenarios. The map was created in ArcGIS 10.3 software (ESRI Inc., Redlands, CA, USA). The source of the base layer shapefiles from the open access platform National platform for Common Geospatial Information Services (www.tianditu.gov.cn).
(TIF)

S5 Fig Spatial dynamics of expansion and contraction: Western versus eastern regions. (a) Contraction of the high-risk area and (b) Expansion of the high-risk area.
(TIF)

S6 Fig Spatial risk dynamics in the western region, dominated by Nileke, Jinghe, and Wusu. (TIF)
