# Supplementary material for: Integrating MaxEnt and copula models to assess plague-associated ecological suitability under climate change in Northern Tianshan, China
Source: PLoS Negl Trop Dis. 2026 Oct 26;20(10):e0014818. doi: 10.1371/journal.pntd.0014818 (PMC13722314; doi:10.1371/journal.pntd.0014818)
Supplement: Supplementary file 1 [file pntd.0014818.s001.pdf]

Table S1 Environmental variables used in the model for *Marmota baibacina* and *Yersinia pestis.*

Variable	Description	Unit	*Marmota baibacina*	*Yersinia pestis*
Bio1	Annual mean temperature	°C	no	no
Bio2	Mean diurnal range (mean of monthly (max temp - min temp))	°C	no	yes
Bio3	Isothermality (bio2/bio7) (* 100)	-	yes	no
Bio4	Temperature seasonality (standard deviation * 100)	°C	no	no
Bio5	Max temperature of warmest month	°C	no	no
Bio6	Min temperature of coldest month	°C	yes	yes
Bio7	Temperature annual range (bio5-bio6)	°C	yes	yes
Bio8	Mean temperature of wettest quarter	°C	no	no
Bio9	Mean temperature of driest quarter	°C	no	no
Bio10	Mean temperature of warmest quarter	°C	no	no
Bio11	Mean temperature of coldest quarter	°C	no	no
Bio12	Annual precipitation	mm	no	no
Bio13	Precipitation of wettest month	mm	no	no
Bio14	Precipitation of driest month	mm	no	no
Bio15	Precipitation seasonality (coefficient of variation)	-	yes	yes
Bio16	Precipitation of wettest quarter	mm	no	no
Bio17	Precipitation of driest quarter	mm	no	no
Bio18	Precipitation of warmest quarter	mm	yes	no
Bio19	Precipitation of coldest quarter	mm	no	no
Elevation	Elevation from the sea level	m	yes	yes

Note: yes indicates factors ultimately included in the model, while no indicates factors not included in the model.
