# Supplementary material for: Integrating MaxEnt and copula models to assess plague-associated ecological suitability under climate change in Northern Tianshan, China
Source: PLoS Negl Trop Dis. 2026 Oct 26;20(10):e0014818. doi: 10.1371/journal.pntd.0014818 (PMC13722314; doi:10.1371/journal.pntd.0014818)
Supplement: Supplementary file 2 [file pntd.0014818.s002.pdf]

Table S2 Four emission scenarios in the shared socioeconomic pathway scenario.

Climatic scenario	Description
SSP1-2.6	Low emission scenario: Global CO_2 emissions will be significantly reduced, reaching net zero after 2050. By 2100, the average temperature will rise by approximately 1.8°C.
SSP2-4.5	Medium emission scenario: Global CO_2 emissions will hover around current levels before starting to decline in the middle of this century, but will not reach net zero by 2100. By 2100, the average temperature will rise by around 2.7°C
SSP3-7.0	High emission scenario: Global CO_2 emissions continue to increase. By 2100, the average temperature will rise by approximately 3.6°C.
SSP5-8.5	Higher emission scenario: Global CO_2 emissions will roughly double by 2050. By 2100, the average temperature will rise by around 4.4°C.
