# Supplementary material for: Integrating MaxEnt and copula models to assess plague-associated ecological suitability under climate change in Northern Tianshan, China
Source: PLoS Negl Trop Dis. 2026 Oct 26;20(10):e0014818. doi: 10.1371/journal.pntd.0014818 (PMC13722314; doi:10.1371/journal.pntd.0014818)
Supplement: Supplementary file 3 [file pntd.0014818.s003.pdf]

Table S3 Percentage contribution of 20 environmental variables in the MaxEnt model for *Marmota baibacina* and *Yersinia pestis*.

Variable	*Marmota baibacina*		*Yersinia pestis*	
	Percentage of variance (%)	Cumulative percentage (%)	Percentage of variance (%)	Cumulative percentage (%)
Bio1	1.1*	3.1	0.8	0.8
Bio2	0.9	1.4	9.9*	1.8
Bio3	3.2*	7.1	0.5	1.0
Bio4	1.4*	11.8	0.6	9.4
Bio5	0.2	1.0	0.0	0.0
Bio6	21.2*	15.3	9.8*	4.3
Bio7	10.5*	16.3	11.6*	36.4
Bio8	1.3*	8.8	0.1	0.1
Bio9	0.2	1.5	0.0	0.2
Bio10	0.1	0.2	0.0	0.1
Bio11	0.3	0.0	0.3	0.1
Bio12	0.4	4.4	0.2	2.8
Bio13	0.0	0.0	0.0	0.0
Bio14	0.3	1.1	0.2	1.7
Bio15	15.2*	6.3	41.2*	29.1
Bio16	2.2*	2.2	0.3	0.6
Bio17	0.4	1.4	0.0	0.0
Bio18	31.4*	14.6	8.5*	9.2
Bio19	2.5*	1.1	0.4	0.7
Elevation	7.1*	2.4	15.5*	1.7

Note: *Variables marked with an asterisk were retained for subsequent spearman correlation analysis.
