# Supplementary material for: Integrating MaxEnt and copula models to assess plague-associated ecological suitability under climate change in Northern Tianshan, China
Source: PLoS Negl Trop Dis. 2026 Oct 26;20(10):e0014818. doi: 10.1371/journal.pntd.0014818 (PMC13722314; doi:10.1371/journal.pntd.0014818)
Supplement: Supplementary file 4 [file pntd.0014818.s004.pdf]

Table S4. Post-hoc variance inflation factor (VIF) assessment of environmental variables following Spearman-based selection for *M. baibacina* and *Y. pestis* MaxEnt models.

**Species**	**Variable**	**VIF**	**Collinearity Status**
M. baibacina	Bio3	2.971	VIF < 10
	Bio6	1.126	VIF < 10
	Bio7	4.348	VIF < 10
	Bio15	2.282	VIF < 10
	Bio18	> 10	High VIF; retained per ecological justification
	Elevation	> 10	High VIF; retained per ecological justification
Y. pestis	Bio2	2.872	VIF < 10
	Bio6	1.235	VIF < 10
	Bio7	1.807	VIF < 10
	Bio15	2.893	VIF < 10
	Elevation	> 10	High VIF; retained per ecological justification
