# Supplementary material for: Integrating MaxEnt and copula models to assess plague-associated ecological suitability under climate change in Northern Tianshan, China
Source: PLoS Negl Trop Dis. 2026 Oct 26;20(10):e0014818. doi: 10.1371/journal.pntd.0014818 (PMC13722314; doi:10.1371/journal.pntd.0014818)
Supplement: Supplementary file 5 [file pntd.0014818.s005.pdf]

Table S5 Average AUC values of training data for MaxEnt models optimized under current and future climate change scenarios.

Species	Item	Current	SSP1-2.6			SSP2-4.5			SSP3-7.0			SSP5-8.5		
			2050s	2070s	2090s	2050s	2070s	2090s	2050s	2070s	2090s	2050s	2070s	2090s
Marmota baibacina	AUC values	0.953	0.955	0.955	0.955	0.957	0.955	0.956	0.954	0.955	0.954	0.955	0.953	0.955
	standard deviation	0.002	0.003	0.002	0.003	0.002	0.002	0.003	0.002	0.002	0.002	0.001	0.003	0.003
Yersinia pestis	AUC values	0.962	0.961	0.955	0.960	0.958	0.963	0.959	0.955	0.958	0.958	0.954	0.960	0.958
	standard deviation	0.009	0.009	0.012	0.007	0.010	0.007	0.011	0.008	0.008	0.007	0.008	0.012	0.007
