# Supplementary material for: Integrating MaxEnt and copula models to assess plague-associated ecological suitability under climate change in Northern Tianshan, China
Source: PLoS Negl Trop Dis. 2026 Oct 26;20(10):e0014818. doi: 10.1371/journal.pntd.0014818 (PMC13722314; doi:10.1371/journal.pntd.0014818)
Supplement: Supplementary file 6 [file pntd.0014818.s006.pdf]

Table S6 The changes of key environmental factor in regions with high suitable habitats for *Marmota baibacina* and *Yersinia pestis* under future climate change.

scenarios	Year	*Marmota baibacina*						*Yersinia pestis*			
		Bio6		Bio7		Bio15		Bio15		Elevation	
		Range	Mean ± SD	Range	Mean ± SD	Range	Mean ± SD	Range	Mean ± SD	Range	Mean ± SD
	Current	-24.87 to -17.82	-22.12 ± 2.06	40.13 to 43.40	41.76 ± 0.96	66.98 to 81.48	74.23 ± 4.23	63.46 to 77.78	70.62 ± 4.18	1420.64 to 2444.20	1932.64 ± 297.90
SSP1-2.6	2050	-24.59 to -17.79	-21.92 ± 2.03	39.98 to 43.17	41.58 ± 0.93	66.69 to 81.70	74.19 ± 4.38	62.75 to 77.96	70.36 ± 4.44	1448.28 to 2445.40	1946.84 ± 290.41
	2070	-24.67 to -17.64	-21.88 ± 2.13	39.86 to 43.34	41.60 ± 1.02	66.85 to 81.72	74.28 ± 4.34	63.01 to 77.86	70.43 ± 4.33	1407.62 to 2414.95	1911.29 ± 293.12
	2090	-24.87 to -17.70	-22.00 ± 2.15	40.19 to 43.34	41.76 ± 0.92	66.80 to 81.57	74.18 ± 4.31	63.17 to 77.94	70.56 ± 4.31	1450.74 to 2428.18	1939.46 ± 284.47
SSP2-4.5	2050	-24.75 to -17.77	-22.06 ± 2.04	40.01 to 43.31	41.66 ± 0.96	66.94 to 81.70	74.32 ± 4.31	62.90 to 78.01	70.46 ± 4.41	1420.35 to 2410.49	1915.42 ± 288.21
	2070	-24.64 to -17.56	-21.80 ± 2.17	40.15 to 43.24	41.69 ± 0.90	66.80 to 81.55	74.17 ± 4.30	63.78 to 78.22	71.00 ± 4.21	1462.51 to 2393.36	1927.94 ± 271.10
	2090	-24.86 to -17.84	-22.16 ± 2.02	39.87 to 43.22	41.55 ± 0.98	65.89 to 81.67	73.78 ± 4.60	63.32 to 78.04	70.68 ± 4.29	1448.07 to 2401.35	1924.71 ± 277.49
SSP3-7.0	2050	-24.84 to -17.77	-22.10 ± 2.07	39.77 to 43.25	41.51 ± 1.01	66.75 to 81.90	74.32 ± 4.42	63.09 to 78.29	70.69 ± 4.43	1433.15 to 2430.79	1931.97 ± 290.35
	2070	-24.91 to -17.61	-21.95 ± 2.21	40.01 to 43.12	41.56 ± 0.91	67.05 to 81.46	74.25 ± 4.21	63.70 to 78.22	70.96 ± 4.24	1433.39 to 2416.00	1924.69 ± 286.01
	2090	-24.79 to -17.75	-22.07 ± 2.06	39.88 to 43.28	41.58 ± 0.99	66.81 to 81.90	74.36 ± 4.40	62.99 to 77.99	70.49 ± 4.37	1452.24 to 2406.75	1929.50 ± 278.02

SSP5-8.5	2050	-24.83 to -17.75	-22.07 ± 2.08	39.82 to 43.34	41.58 ± 1.03	66.68 to 81.87	74.27 ± 4.43	62.88 to 78.30	70.59 ± 4.50	1391.93 to 2426.90	1909.41 ± 301.19
	2070	-24.56 to -17.74	-21.84 ± 2.07	40.09 to 43.23	41.66 ± 0.92	66.67 to 81.56	74.12 ± 4.34	63.61 to 77.58	70.60 ± 4.08	1420.79 to 2356.11	1888.45 ± 272.40
	2090	-24.92 to -17.76	-22.13 ± 2.09	40.02 to 43.12	41.57 ± 0.91	66.71 to 81.59	74.15 ± 4.34	63.22 to 78.05	70.63 ± 4.33	1417.16 to 2388.58	1902.87 ± 282.88
