# Supplementary material for: Integrating MaxEnt and copula models to assess plague-associated ecological suitability under climate change in Northern Tianshan, China
Source: PLoS Negl Trop Dis. 2026 Oct 26;20(10):e0014818. doi: 10.1371/journal.pntd.0014818 (PMC13722314; doi:10.1371/journal.pntd.0014818)
Supplement: Supplementary file 7 [file pntd.0014818.s007.pdf]

Table S7 The areas of potential distribution regions with high transmission risk for plague under current and future climate conditions in the focus.

Region	Scenarios	Periods	High risk areas ($\times 10^4 km^2$)	Stable ($\times 10^4 km^2$)	Contraction ($\times 10^4 km^2$)	Expansion ($\times 10^4 km^2$)	Percentage stable (%)	Percentage contraction (%)	Percentage expansion (%)
Western		Current	0.31						
	SSP1-2.6	2050S	0.25	0.12	0.19	0.13	38.71	61.29	41.94
		2070S	0.23	0.15	0.16	0.08	48.39	51.61	25.81
		2090S	0.28	0.17	0.14	0.11	54.84	45.16	35.48
	SSP2-4.5	2050S	0.26	0.12	0.19	0.14	38.71	61.29	45.16
		2070S	0.36	0.18	0.13	0.18	58.06	41.94	58.06
		2090S	0.25	0.07	0.24	0.18	22.58	77.42	58.06
	SSP3-7.0	2050S	0.29	0.16	0.15	0.13	51.61	48.39	41.94
		2070S	0.24	0.10	0.21	0.14	32.26	67.74	45.16
		2090S	0.30	0.12	0.19	0.18	38.71	61.29	58.06
	SSP5-8.5	2050S	0.32	0.15	0.16	0.17	48.39	51.61	54.84
		2070S	0.40	0.16	0.15	0.24	51.61	48.39	77.42
		2090S	0.35	0.07	0.24	0.28	22.58	77.42	90.32
Eastern		Current	0.17						
	SSP1-2.6	2050S	0.19	0.12	0.05	0.07	70.59	29.41	41.18
		2070S	0.16	0.13	0.04	0.03	76.47	23.53	17.65
		2090S	0.21	0.15	0.02	0.06	88.24	11.76	35.29
	SSP2-4.5	2050S	0.15	0.11	0.06	0.04	64.71	35.29	23.53
		2070S	0.18	0.13	0.04	0.05	76.47	23.53	29.41
		2090S	0.14	0.10	0.07	0.04	58.82	41.18	23.53

SSP3-7.0	2050S	0.18	0.13	0.04	0.05	76.47	23.53	29.41
	2070S	0.34	0.16	0.01	0.18	94.12	5.88	105.88
	2090S	0.16	0.12	0.05	0.04	70.59	29.41	23.53
SSP5-8.5	2050S	0.20	0.14	0.03	0.06	82.35	17.65	35.29
	2070S	0.17	0.13	0.04	0.04	76.47	23.53	23.53
	2090S	0.20	0.11	0.06	0.09	64.71	35.29	52.94

Note: Percentage stable, contraction, and expansion values are calculated as the area proportion relative to relative to the current high-risk plague zone area.
